# Effectiveness, safety and pharmacokinetics of Polo-like kinase 1 inhibitors in tumor therapy: A systematic review and meta-analysis

**DOI:** 10.3389/fonc.2023.1062885

**Published:** 2023-02-09

**Authors:** Xiao Wei, Mingzhu Song, Chan Huang, Qiao Yu, Guirong Jiang, Guanghao Jin, Xibiao Jia, Zheng Shi

**Affiliations:** ^1^ School of Preclinical Medicine, Chengdu University, Chengdu, China; ^2^ Key Laboratory of Ministry of Education of Birth Defects and Related Maternal and Child Diseases, West China Second Hospital, Sichuan University, Chengdu, China; ^3^ Clinical Genetics Laboratory, Clinical Medical College and Affiliated Hospital of Chengdu University, Chengdu University, Chengdu, China

**Keywords:** effectiveness, safety, pharmacokinetics, PLK1 inhibitors, tumor therapy

## Abstract

**Objective:**

To provide a systematic review of existing meta-analysis on the efficacy, safety and pharmacokinetics of the novel Polo-like kinase-1 (Plk1) inhibitors in various tumor treatments, and assess the methodological quality and the strength of evidence of the included meta-analysis.

**Methods:**

The Medline, PubMed, Embase, etc. were searched and updated on 30 June 2022. 22 eligible clinical trials involving a total of 1256 patients were included for analyses. Randomised controlled trials (RCTs) compared the efficacy or safety, or both of any Plk1 inhibitors with placebo (active or inert) in participants. To be included, studies had to be RCTs, quasi-RCTs, and nonrandomized comparative studies.

**Results:**

A meta-analysis of two trials reported progression-free survival (PFS) of the overall population (effect size (ES), 1.01; 95% confidence intervals (CIs), 0.73-1.30, *I^2^ =*0.0%, *P*<0.001) and overall survival (OS) of the overall population (ES, 0.91; 95% CIs, 0.31-1.50, *I^2^ =*77.6%, *P*=0.003). 18 adverse events (AEs) reflected that the possibility of occurrence of AEs in the Plk1 inhibitors group was 1.28 times higher than in the control group (odds ratios (ORs), 1.28; 95% CIs,1.02-1.61). The results of meta-analysis showed that the incidence of AEs in the nervous system was the highest (ES, 0.202; 95% CIs, 0.161-0.244), followed by blood system (ES, 0.190; 95% CIs, 0.178-0.201) and digestive system (ES, 0.181; 95% CIs, 0.150-0.213). Rigosertib (ON 01910.Na) was associated with a decreased risk of AEs in digestive system (ES, 0.103; 95% CIs, 0.059-0.147), but BI 2536 and Volasertib (BI 6727) increased risk of AEs in blood system (ES, 0.399; 95% CIs, 0.294-0.504). Five eligible studies reported the pharmacokinetic parameters of the low dosage (100 mg) cohort and the high dosage (200 mg) cohort, and there was no statistical difference in the total plasma clearance, terminal half-life and apparent volume of distribution at steady state.

**Conclusions:**

Plk1 inhibitors work better in improving OS and they are well tolerated, effective and safe in reducing the severity of illness while improving the quality of life, especially in patients with non-specific tumors, respiratory system tumors, musculoskeletal system tumors, and urinary system tumors. However, they fail to prolong the PFS. From the vertical whole level analysis, compared to other systems in the body, Plk1 inhibitors should be avoided as far as possible for the treatment of tumors related to the blood circulatory system, digestive system and nervous system, which were attributed to the intervention of Plk1 inhibitors associated with an increased risk of AEs in these systems. The toxicity caused by immunotherapy should be carefully considered. Conversely, a horizontal comparison of three different types of Plk1 inhibitors suggested that Rigosertib (ON 01910.Na) might be relatively suitable for the treatment of tumors associated with the digestive system, while Volasertib (BI 6727) might be even less suitable for the treatment of tumors associated with the blood circulation system. Additionally, in the dose selection of Plk1 inhibitors, the low dose of 100 mg should be preferred, and meanwhile, it can also ensure the pharmacokinetic efficacy that is indistinguishable from the high dose of 200 mg.

**Systematic review registration:**

https://www.crd.york.ac.uk/prospero/, identifier CRD42022343507.

## Introduction

Many advanced or metastatic human cancers are incurable despite the availability of a variety of conventional treatment modalities, mainly including surgery (1), chemotherapy, radiation therapy, immunotherapy (2), or combinations of these (3). Objective responses in patients with advanced disease, though frequently seen when using conventional treatments, are often followed by tumor progression and death (4). Therefore, the exploration for new therapeutic strategies has become an urgent priority.

The understanding of cell cycle regulation in cancer biology has increased considerably in recent years. The Polo family of serine/threonine protein kinases is highly conserved in all eukaryotes and has been identified as important regulators of cell division and its checkpoints (5, 6). As a member of the family, Polo-like kinase-1 (Plk1) possesses two highly conserved functional domains that can serve as the potential target sites (7, 8). They can regulate several important steps during mitosis, including mitotic entry, centrosome maturation and separation, formation of the bipolar spindle, metaphase to anaphase transition, and initiation of cytokinesis. Specifically, the steps involved by Plk1 mainly include the initiation of cell mitosis, centrosome separation and maturation, the transition from metaphase to anaphase and mitotic exit, and the onset of allowing cell division. Accordingly, the inhibition of Plk1 can lead to a disruption in spindle assembly, which further results in a distinct mitotic arrest phenotype and subsequent apoptosis (9, 10). Furthermore, although Plk1 is active during mitosis, it does not seem to work in non-dividing cells (11, 12).

To our best knowledge, the overexpression of Plk1 observed in several human cancers, such as non-small cell lung cancer (NSCLC) (13, 14) and pancreatic cancer (15), is closely associated with poor prognosis and inferior overall survival (9–11, 16). Moreover, it has also been found that Plk1 overexpression in approximately 80% of human tumors is associated with an upregulated poor prognosis in malignancies with high mitotic activity (17), such as NSCLC (11). The majority of these functions are mainly attributed to the Plk1, that is regarded as the most extensively characterized mammalian polo-like kinase (12, 18).

In the above context, a number of the inhibitors related to the Plk1 pathway or relevant modulators has been born and currently in early clinical development (16). Accordingly, Plk1 inhibitors may represent a new promising therapeutic approach with a novel mode of action in oncology, and several Plk1 inhibitors (Rigosertib/ON 01910.Na, Volasertib/BI 6727, BI 2536, GSK461364A) have been investigated in preclinical studies (6, 8, 17, 19, 20). Even so, Plk1 inhibitors have attracted controversy as to whether they are effective and safe anti-tumor agents. To solve the puzzle, here we set out to make a systematic review and meta-analysis of RCTs to gain insight relative limitation and benefits of Plk1 inhibitors in patients with cancer.

## Material and methods

The systematic review and meta-analysis were performed in accordance with the Preferred Reporting Items for Systematic Reviews and Meta-Analyses (PRISMA) guidelines. The research protocol was registered and approved in PROSPERO (registration # CRD42022343507).

### Data sources and searches

The search was conducted in accordance with the principles outlined in the Cochrane Handbook for Systematic Reviews of Interventions (21). Studies were identified by searching electronic databases and relevant websites. We carried out a comprehensive search to identify potential articles in PUBMED, MEDLINE database and EMBASE up to June 2022, by using the search terms: “Polo-like Kinase 1 Inhibitor” or “Plk1 inhibitor” or “Rigosertib” or “ON 01910.Na” or “Volasertib” or “BI 2536” or “BI 6727” or “GSK461364A” and “cancer” or “tumor” or “carcinoma” or “neoplasm”, limited to clinical trials. There was no limit on the language of publication. In order to ensure the completeness and quality of the results, relevant scientific meetings were retrieved and independent search was performed by using the Web of Science database, and unpublished trials were checked in the clinical trial registry (http://www.clinicaltrials.gov).

### Selection criteria and data extraction

The search was complemented by additional sources, including relevant systematic reviews and the reference lists of included studies that were manually searched to identify additional potentially relevant studies. To be included, studies had to be RCTs, quasi-RCTs, and nonrandomized comparative studies, and had to report at least one outcome of interest (4). Secondary outcomes included AEs that were reported individually or collectively (i.e., based on severity grading systems). Single-arm trials and trials with Plk1 inhibitors used in both arms were accepted. Studies with no comparative elements were excluded. Trials with Plk1 inhibitors used to treat other diseases were also excluded. In all the included RCTs, Plk1 inhibitors were used alone or in combination with other chemotherapeutic agents as the treatment group, while placebo or other chemotherapeutic agents were used in the control group. Two investigators independently reviewed the articles to exclude irrelevant and overlapping studies. Disagreement was resolved by discussion, and where no agreement was reached, a third independent party acted as an arbiter.

A data extraction form was first developed specifically to collect information on study design, characteristics of participants, characteristics of interventions, and outcome measures. Two reviewers independently extracted data related to the prespecified outcomes. Primary study authors were asked to provide missing, ambiguous, or important additional data. Here, we collected the following information from all the included RCTs: first author’s surname, year of publication, number of participants, type of tumors, trial phase, type of inhibitors, median age, and male (%). In addition, the ORs and ES of the AEs, and the standard mean difference (SMD) of the pharmacokinetic parameter (22) (e.g., total body clearance rate, terminal half-life, apparent volume of distribution at steady state) with 95% CIs were extracted from most of the trials to evaluate the curative effect of Plk1 inhibitors. Information on AEs was also retrieved to calculate the safety of Plk1 inhibitors (23). A study was not included in the meta-analysis if there was evidence of severe bias or heterogeneity.

### Quality assessment

The quality of the selected studies was evaluated with a methodology checklist developed by the Scottish Inter-collegiate Guidelines Network (SIGN: http://www.sign.ac.uk/pdf/sign50.pdf). Because some studies selected were nonrandomized, we used the SIGN guideline for quality assessment of nonrandomized studies, which consisted of the following judgments: focused questions, selection bias of subjects, assessment of outcomes and exposure status, handling confounders, and provision of confidence intervals.

### Data synthesis and statistical analysis

All analyses were performed with Stata version 13.0 (Stata Corp, College Station, Texas). For data analysis, descriptive statistics was used to summarize the data of baseline characteristics. A quantitative synthesis (i.e., meta-analysis) was performed only in RCTs, and only if methodologically appropriate. The random-effects model was legitimately used due to the anticipated clinical heterogeneity of participants and interventions. For time-to-event data such as PFS and OS, ES and 95% CIs directly obtained from studies were used to compare results by using the inverse variance method. A 2-tailed *P* value of less than 0.05 was judged as statistical significance. ORs and 95% CIs were used to assess the AEs between Plk1 inhibitors group and the control group. In addition, we extracted dichotomous data from all studies reporting number of patients with AEs and total participants, which were pooled for calculating ES with 95% CIs. For the continuous outcomes such as pharmacokinetic parameter, the SMD was calculated. The degree of heterogeneity was measured by the *I^2^* statistic, with *I^2^* < 25%, 25-75% and > 75% to represent low, moderate and high degree of inconsistency, respectively (24). Statistical heterogeneity was defined as an *I^2^* statistic value of more than 50% (24). In analyses, if the heterogeneity was low, then we needed to use a fixed-effect model, or else apply the random-effect model. We further performed a subgroup analysis based on tumor types and different kinds of Plk1 inhibitors (BI 2536, Rigosertib and Volasertib). Funnel plot and Egger’s regression asymmetry test were used to access the publication bias of literatures.

## Results

### Literature search and study characteristics

**Figure 1:**
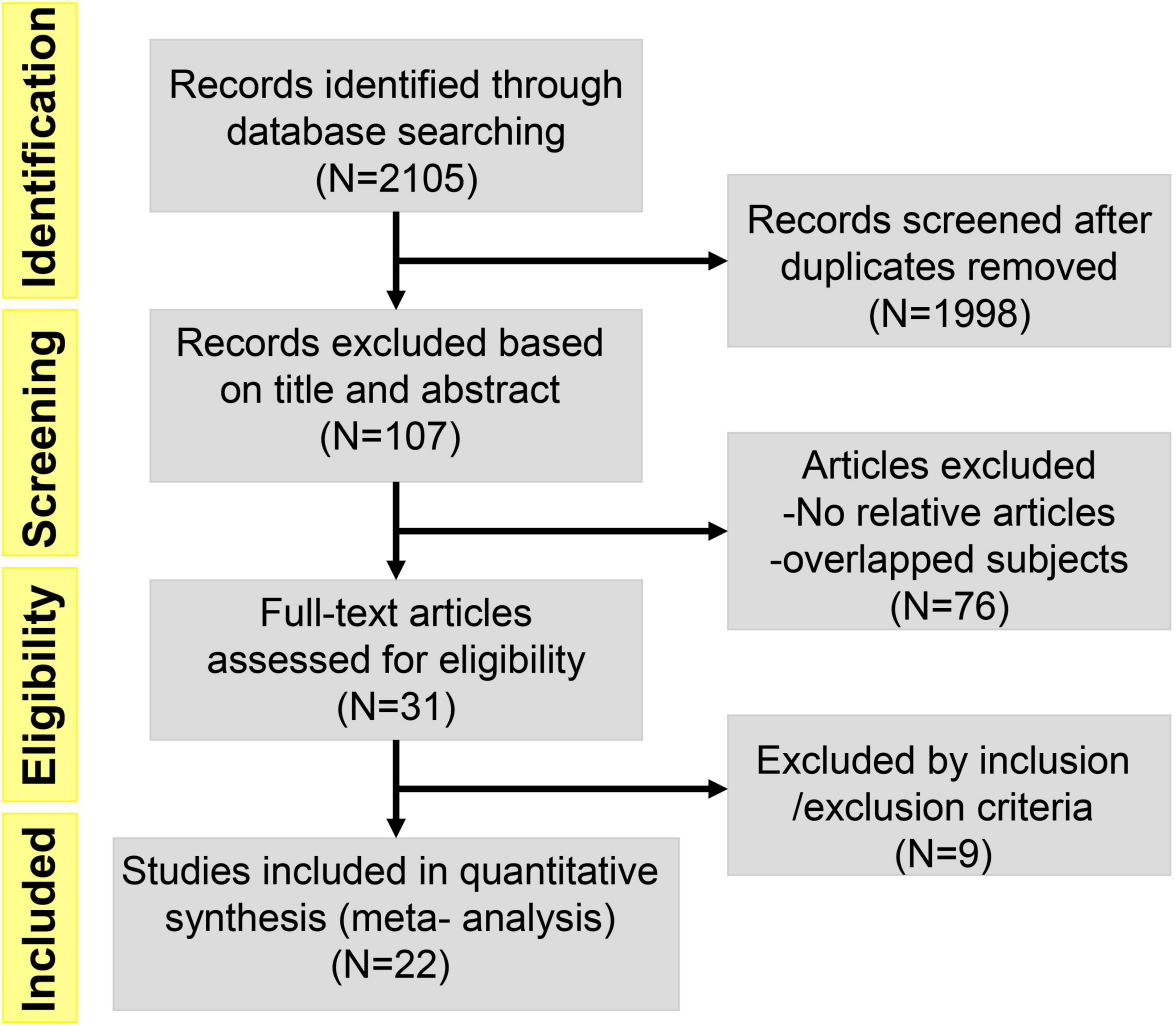
Flow diagram of the literature search and trial selection process (N = number of published papers at each step).

A Preferred Reporting Items for Meta-Analyses (PRIMAs) studies were used in this work. We initially identified 107 potential eligible studies through title and abstract screening. Among them, 76 studies were excluded since they were not relevant to our analysis, leaving 31 articles for full review. After assessing the full texts of these potentially relevant studies, 9 studies were excluded for the following reasons: 14 contained no relative outcomes and 12 were duplicate publication. Ultimately, 22 eligible clinical trials involving a total of 1256 patients were included for analyses. A flow diagram of the trial selection process was showed in **Figure 1**. Data on trial details (e.g., first author, publication year, phase, type of tumors, type of inhibitors, number of patients, and patient characteristics), treatments, and outcomes were separately extracted into a spreadsheet. Survival data assessed by the independent review facility were preferably extracted to avoid potential assessment bias by investigators. We minimised issues arising from potential lack of transitivity by only including RCTs with strict patient allocation, and optimized balance to address all treatments for the same condition. Transitivity was evaluated by the use of descriptive statistics for study and population baselines, such as sample size, age and sex. Age of Participants recruited in different studies ranged from minimum of 17 years to maximum of 87 years, with a median age size of 61. Although the potential eligible studies from EMBASE were partly overlapped with previous publications, some of them provided elaborated incidence about AEs. They were not mentioned in previous meta-analysis-based articles, so these relevant studies were also included. We preferred the use of treatment related AEs, but if not specified as treatment related, we used all adverse events. ClinicalTrials.gov and other available sources were evaluated for the most recent and complete data. Our study included 6 phase II/III clinical trials and 16 phase I clinical trials, and the types of inhibitors involved BI 2536, Volasertib (BI 6727), Rigosertib (ON 01910.Na) and GSK461364A. In all 22 clinical trials, 1 used GSK461364A, 7 used BI 2536, 4 used Rigosertib (ON 01910.Na), and 10 used Volasertib (BI 6727). Moreover, 2 trials were about acute myeloid leukemia (AML), 1 trial was about locally advanced or metastatic urothelial cancer (LAMUC), 1 trial was about non-Hodgkin lymphoma, and 18 trials were about advanced solid tumors. The characteristics of 22 trials included were detailedly presented in **Table 1**.

**Table 1:** Characteristics of the clinical trials included in the meta-analysis.

Authors	Year	Phase	Type of tumors	Type of Inhibitors	Total Sample (N=Test/N=control)	Age in years, Median (range) (N=Test/N=control)	Male, (%) (N=Test/N=control)
Hartmut (19)	2014	II	Non-solid	Volasertib	42/45	75 (65-87)/76 (57-86)	23 (55)/25 (56)
Peter (25)	2015	II	Solid	Volasertib	96/47	NA/62 (44-79)	48 (96)/22 (47)
B. H. O’Neil (26)	2015	II/III	Solid	Rigosertib	106/54	63.2 (29-83)/61.8 (30-87)	69 (65)/31 (57)
Julie M (27)	2013	I	Non-solid	BI 2536	17/24	64.0 (38-74)/69.5 (22-87)	9 (53)/10 (42)
Walter M (28)	2014	II	Non-solid	Volasertib	50/NA	68.5 (52–83)/NA	40 (80)/NA
Patrick Scho¨ffski (9)	2011	I	Solid	BI 6727	65/NA	58 (19-79)/NA	38 (58)/NA
Martin Sebastian (5)	2010	II	Solid	BI 2536	48/47	64 (42-80)/65 (38-81)	33 (69)/34 (72)
F.de Braud (29)	2015	I	Solid	Volasertib	30/NA	56.5 (33-74)/NA	18 (60)/NA
Ahmad Awada (11)	2015	I	Solid	Volasertib	30/31	55 (17-77)/58 (23-81)	16 (53.3)/18 (58.1)
Hiroshi Nokihara (30)	2016	I	Solid	Volasertib	15/NA	64 (39-75)/NA	13 (86.7)/NA
Yukio Kobayashi (18)	2015	I	Non-solid	Volasertib	19/NA	73 (53-86)/NA	7 (36.8)/NA
Jean-Pascal Machiels (31)	2015	I	Solid	Volasertib	29/28	54.0 (38-77)/63.5 (47-81)	14 (48.3)/15 (53.6)
C-C Lin (32)	2014	I	Solid	BI 6727	32/27	53.5 (37-78)/58.0 (31-77)	20 (62.5)/15 (55.6)
A.Frost (33)	2012	I	Solid	BI 2536	21/NA	61 (33-75)/NA	12 (57.1)/NA
Wen Wee Ma (15)	2012	I	Solid	Rigosertib	40/NA	57 (26-80)/NA	14 (35)/NA
Daniel W (34).	2014	I	Solid	ON 01910.Na	48/NA	NA (20-79)/NA	24 (50)/NA
Antonio Jimeno (35)	2008	I	Solid	ON 01910.Na	20/NA	63 (46-73)/NA	9 (45)/NA
David Olmos (8)	2011	I	Solid	GSK461364A	23/17	62.0 (31-80)/62.0 (28-75)	13 (57)/12 (71)
Peter M.Ellis (36)	2013	I	Solid	BI 2536	41/NA	59.0 (42-79)/NA	23 (56)/NA
Klaus Mross (37)	2008	I	Solid	BI 2536	40/NA	61.0 (37-75)/NA	21 (52.5)/NA
Ralf-Dieter Hofheinz (38)	2010	I	Solid	BI 2536	44/26	65 (36-77)/63 (43-84)	30 (68.2)/18 (69.2)
Patrick Scho¨ffski (10)	2010	II	Solid	BI 2536	71/NA	57.7/NA	27 (38.0)/NA

NA, not available; N, number of patient demographic.

### Outcomes

#### Efficacy

The efficacy of the Plk1 inhibitors group and the control group for tumors was evaluated by combining PFS and OS. We included these two studies to analyze PFS and OS, respectively. Two trials reported PFS of the overall population (ES, 1.01; 95% CIs, 0.73-1.30) (**Table 2** and **Figure S1**). There was very slight heterogeneity between these two studies, so the random effect model could be used. According to the forest plot presented, PFS was 1.01 (*I^2^* = 0.0%, *Z* = 6.98, *P* < 0.001). Assume that the test confirms that the difference was statistically significant, suggesting that the Plk1 inhibitors group failed to prolong the PFS. In addition, two trials reported OS of the overall population (ES, 0.91; 95% CIs, 0.31-1.50) (**Table 2** and **Figure S2**). There was obviously heterogeneity between both studies, so the random effect model should be used. As the pooled ES exhibited, OS was 0.91 (*I^2^* =77.6%, *Z* = 2.98, *P* = 0.003). Assume that the test confirms that the difference was statistically significant, indicating that the OS of the Plk1 inhibitors group was significantly improved in comparison with that of the control group in the overall population.

**Table 2:** The pooled ES for OS and PFS by overall population.

Research index	Authors	Year	HR	Lci	Uci	*I^2^*	Merging method of effects	ES [95% CIs]	*Z*	*P*
OS	Hartmut (19)	2014	0.630	0.400	1.000	77.60%	random-effect model	0.905[0.310-1.500]	2.98	0.003
	B. H. O’Neil (26)	2015	1.240	0.850	1.810					
PFS	Peter (25)	2015	1.140	0.730	1.771	0.00%	random-effect model	1.014 [0.729-1.299]	6.98	<0.001
	B. H. O’Neil (26)	2015	0.960	0.680	1.360					

#### Safety

**Figure 2:**
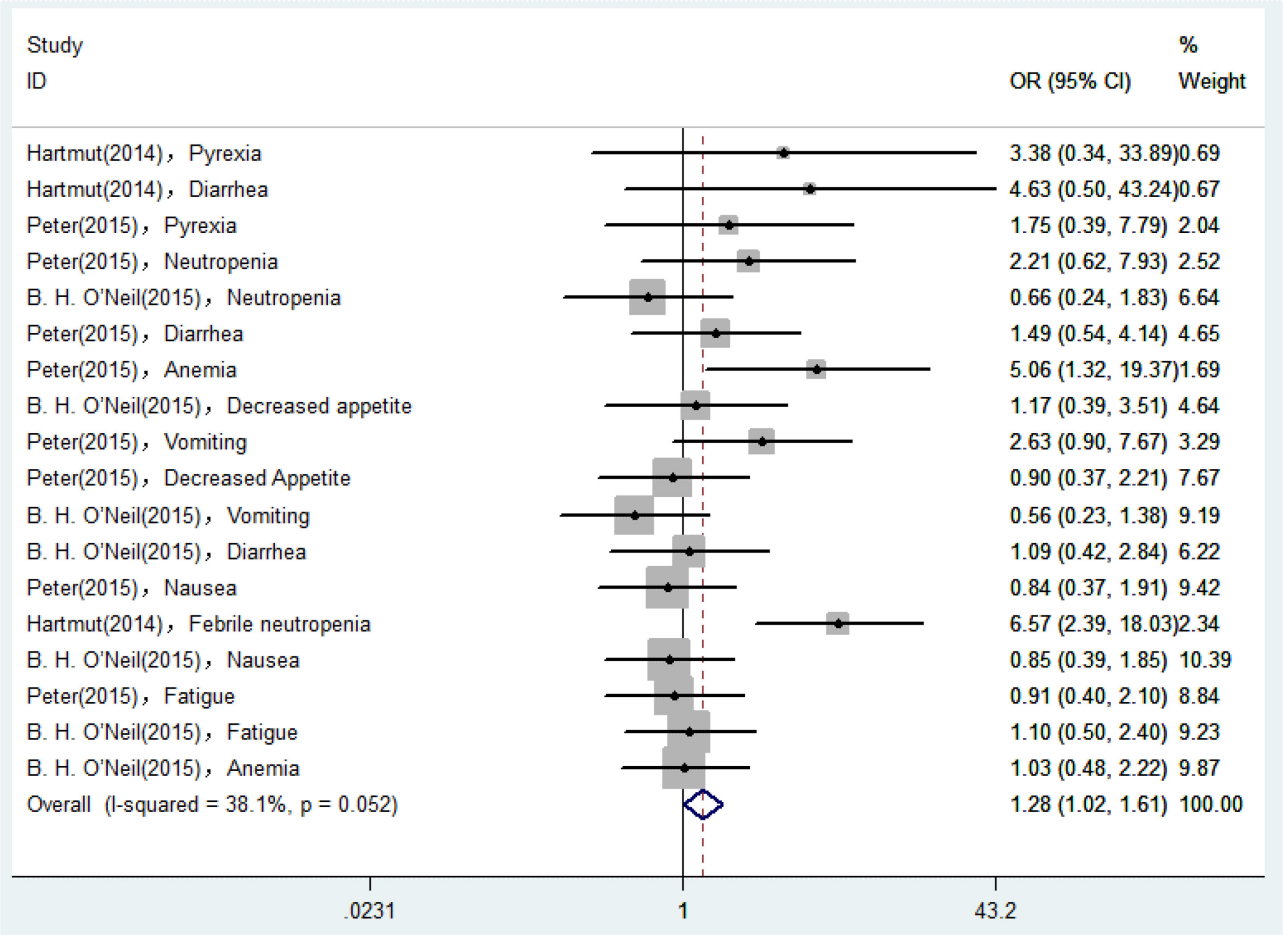
Forest plots of the pooled ORs for incidence of AEs in the RCTs.

18 AEs reported in eligible RCTs were used for analyses, and the degree of heterogeneity was assessed by the *I^2^* statistic. As shown in **Figure 2**, *I^2^* was equal to 38.1%, suggesting that the heterogeneity remained low and the degree was acceptable. Therefore, we could select a fixed-effect model to perform heterogeneity tests for pooled results. According to the pooled ORs for incidence of AEs, the possibility of occurrence of AEs in the Plk1 inhibitors group was 1.28 times higher than that in the control group (ORs, 1.28; 95% CIs, 1.02-1.61). Assume that the test confirms that the difference was statistically significant (*Z* = 2.10, *P* = 0.036).

### Analysis of publication bias

**Figure 3:**
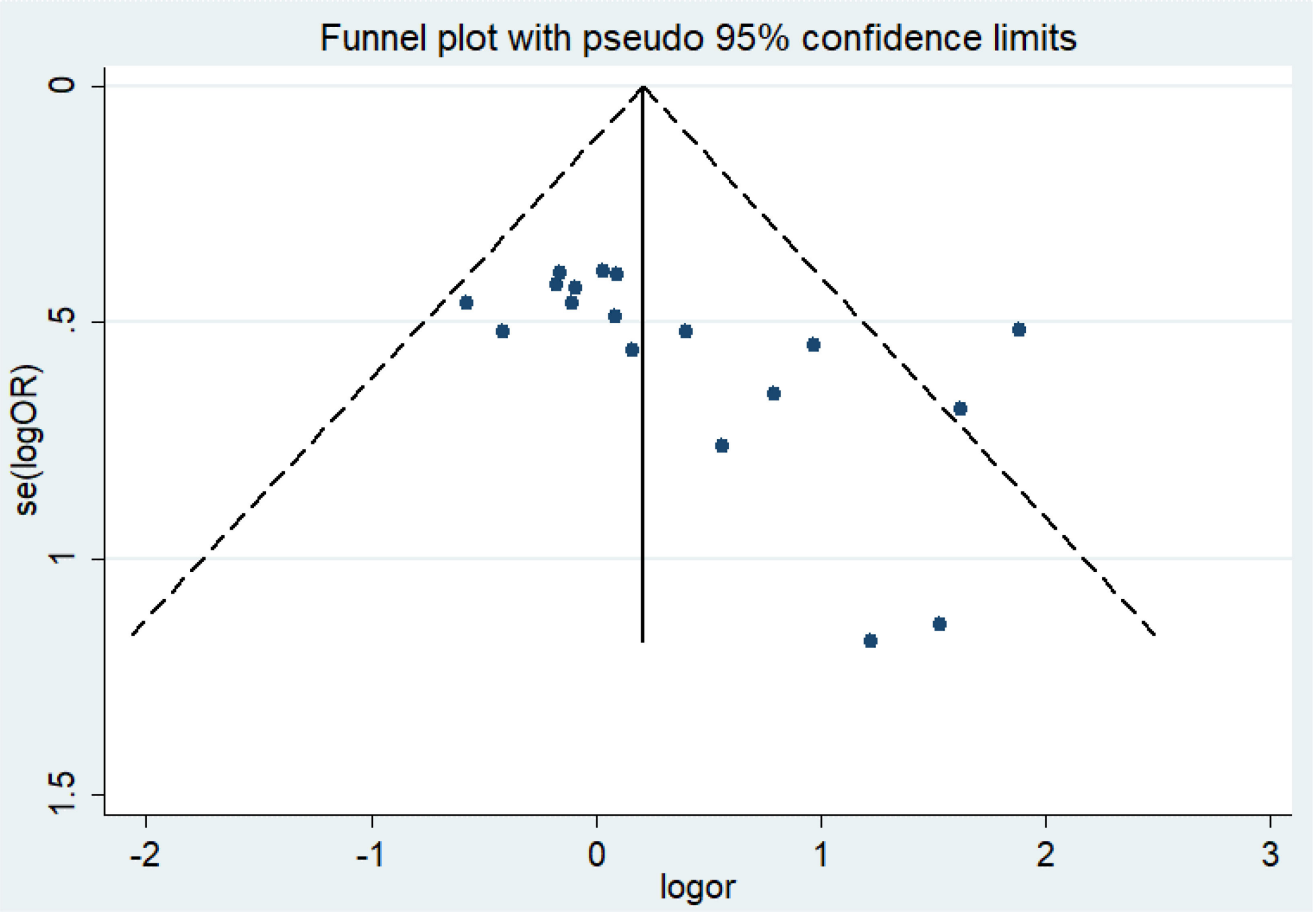
Funnel plot of asymmetry test of the publication bias of literatures.

For the quality assessment of the studies, the bias risk assessment tool recommended by Cochrane is applied to evaluate the quality of all included studies and the risk of bias. The assessment will include random sequence generation, allocation concealment, blinding of participants and personnel, blinding of outcome assessment, incomplete outcome data, selective outcome reporting, and other sources of bias. The risk of high and low bias can be expressed as “high risk” and “low risk”, respectively. The information provided in the study is inaccurate or insufficient for the bias assessment, which can be expressed as “unclear risk”. The evaluation of the above content is independently evaluated by two researchers. If there are different opinions, the discussion will be conducted. If there are still differences, consult the third appraiser. Otherwise, consult with the Cochrane Professional Group. We used Funnel plot and Egger’s regression asymmetry test to access the publication bias of literatures. Arrangement of data points did reveal evidence of obvious asymmetry. It was further confirmed by Egger’s linear regression asymmetry test for each outcome, and the results still did show evidence of publication bias (*t* = 2.70, *P* = 0.016) (**Figure 3** and **Table S1**). Therefore, more studies will be needed to provide stronger evidence in the future.

### Assessment of risk of bias

Three investigators (Hartmut, Peter and B. H. O’Neil) were independently appraised the potential risk of bias of the RCTs by using the Cochrane Risk of Bias Methods at the study and outcome levels. Methodological quality summary presented the authors judgments about each methodological quality item for each included study. Potential risks of bias within individual trials were shown in **Table 3**. The included RCTs were at high risk of bias and unclear methodological quality items. Selection bias, caused by inappropriate methods of generating random sequences, was the main source of potential bias in this meta-analysis.

**Table 3:** The risk of bias of independent small studies.

Authors (Year)	Random sequence generation (Judgment evidence)	Distributive hiding (Judgment evidence)	Blinding (Judgment evidence)	Integrity of outcome data (Judgment evidence)	Selective reporting (Judgment evidence)	Other bias (Judgment evidence)
Hartmut (19) (2014)	High risk of bias (Randomization was not stratified for any patient or disease characteristics.)	Unclear (Hidden method is not described)	Unclear (Blind method is not described)	Low risk of bias (One patient was lost to follow-up after 47 days, the missing data does not affect the analysis of the results.)	Low risk of bias (The trial reported all pre-specified outcomes in a pre-specified manner.)	Unclear (Other possible biases are not described)
Peter (25) 2015)	High risk of bias (Stratified according to cancer type, Eastern Cooperative Oncology Group performance status of 0 to 2)	Unclear (Hidden method is not described)	Unclear (Blind method is not described)	High risk of bias (It was not possible to obtain OS data on patients deceased before this time, resulting in a large number of censored observations)	High risk of bias (OS data are not reported. At the time of data cut off, No CRs were observed)	Unclear (Other possible biases are not described)
B. H. O’Neil (26) (2015)	High risk of bias (The patients were randomized and stratified according to ECOG performance status of ≤2)	Unclear (Hidden method is not described)	Unclear (Blind method is not described)	High risk of bias (Patients were followed until 125 deaths occurred, there remains two patients on treatment)	Low risk of bias (The trial reported all pre-specified outcomes in a pre-specified manner.)	Unclear (Other possible biases are not described)

A total of 22 eligible articles revealed treatment outcomes with Plk1 inhibitors. The type of AEs primarily involved the respiratory system, urinary system, skin system, nervous system, facial features, digestive system (liver function, stomach, nausea or vomiting, appetite, abdomen), hematopoietic system (anemia, neutropenia, leukopenia, thrombocytopenia), musculoskeletal system, and non-specific tissues and organs. In this study, the meta-analysis was used to combine the incidence of AEs in single group, and the heterogeneity analysis and summary analysis were displayed in the **Table 4** and **Figures S3–S18**. From these outcomes, it was clearly observed that the incidence of all AEs was not more than 20.2%. Of which, the incidence of AEs in the nervous system was shown to be the highest (ES, 0.202; 95% CIs, 0.161-0.244), followed by in blood system (ES, 0.190; 95% CIs, 0.178-0.201) and in digestive system (ES, 0.181; 95% CIs, 0.150-0.213). In particular, the incidence of AEs in the non-specific system was only 6.9% (ES, 0.069; 95% CIs, 0.046-0.093).

**Table 4:** The incidence of AEs in the overall estimate with whole body system.

Type of AEs	*I^2^*	Merging method of effects	ES [95% CIs]	*Z*	*P*
Respiratory	79.30%	random-effect model	0.138 [0.049,0.227]	3.030	0.002
Urinary	70.20%	random-effect model	0.109 [0.074,0.143]	6.120	<0.001
Skin	61.80%	random-effect model	0.179 [0.140,0.219]	8.870	<0.001
Nervous	89.70%	random-effect model	0.202 [0.161,0.244]	9.540	<0.001
Facial features	19.10%	fixed-effect model	0.162 [0.125,0.198]	8.620	<0.001
Digestive	88.40%	random-effect model	0.181 [0.150,0.213]	11.330	<0.001
Hematopoietic	92.60%	random-effect model	0.190 [0.178,0.201]	32.160	<0.001
Musculoskeletal	57.10%	random-effect model	0.137 [0.060,0.213]	3.510	<0.001
Non-specific	35.90%	fixed-effect model	0.069 [0.046,0.093]	5.730	<0.001

P, P-value for variation in ES attributable to heterogeneity chi-squared test.

To evaluate the safety of Plk1 inhibitors, we analyzed the risk factor of any side-effects in the overall population with AEs. All included studies concerning the main comparisons of interventions were summarized in **Tables 5**, **6** and **Figures S19–S24**. In this study, the relationship between Plk1 inhibitors and type of AEs was presented here. After the intervention with the Plk1 inhibitors, the group of non-solid tumors presented the highest incidence of AEs in the blood system (ES, 0.426; 95% CIs, 0.117-0.736), while the group of solid tumors showed the lowest incidence of AEs in the digestive system (ES, 0.180; 95% CIs, 0.147-0.212). Furthermore, three Plk1 inhibitors were used primarily in the clinical trials, thus we also separately evaluated the safety of the BI 2536, Volasertib (BI 6727) and Rigosertib (ON 01910.Na) subgroups. Analysis of patients in the overall population showed that, compared with other systems, Rigosertib (ON 01910.Na) were associated with a decreased risk of AEs in digestive system (ES, 0.103; 95% CIs, 0.059-0.147), but Volasertib (BI 6727) increased risk of AEs in blood system (ES, 0.399; 95% CIs, 0.294-0.504).

**Table 5:** The incidence of AEs in the overall estimate with different tumor types.

Type of AEs	Type of tumors					
	Solid			Non-solid		
	ES [95% CIs]	*Z*	*P*	ES [95% CIs]	*Z*	*P*
Nervous	0.201 [0.159,0.244]	9.30	<0.001	0.235 [0.100,0.369]	3.42	0.001
Blood	0.286 [0.235,0.336]	11.00	<0.001	0.426 [0.117,0.736]	2.70	0.007
Digestive	0.180 [0.147,0.212]	10.92	<0.001	0.266 [-0.004,0.536]	1.93	0.054

P, P-value for variation in ES attributable to heterogeneity chi-squared test.

**Table 6:** The incidence of AEs in the overall estimate with different inhibitor types.

Type of AEs	Type of Inhibitors								
	BI 2536			Volasertib (BI 6727)			Rigosertib (ON 01910.Na)		
	ES [95% CIs]	*Z*	*P*	ES [95% CIs]	*Z*	*P*	ES [95% CIs]	*Z*	*P*
Nervous	0.175[0.124,0.226]	6.76	<0.001	0.239[0.159,0.319]	5.84	<0.001	0.179[0.074,0.284]	3.34	0.001
Blood	0.215[0.172,0.258]	9.80	<0.001	0.399[0.294,0.504]	7.46	<0.001	0.152[0.097,0.208]	5.37	<0.001
Digestive	0.202[0.144,0.260]	6.78	<0.001	0.213[0.156,0.270]	7.38	<0.001	0.103[0.059,0.147]	4.56	<0.001

P, P-value for variation in ES attributable to heterogeneity chi-squared test.

### Pharmacokinetics

**Figure 4:**
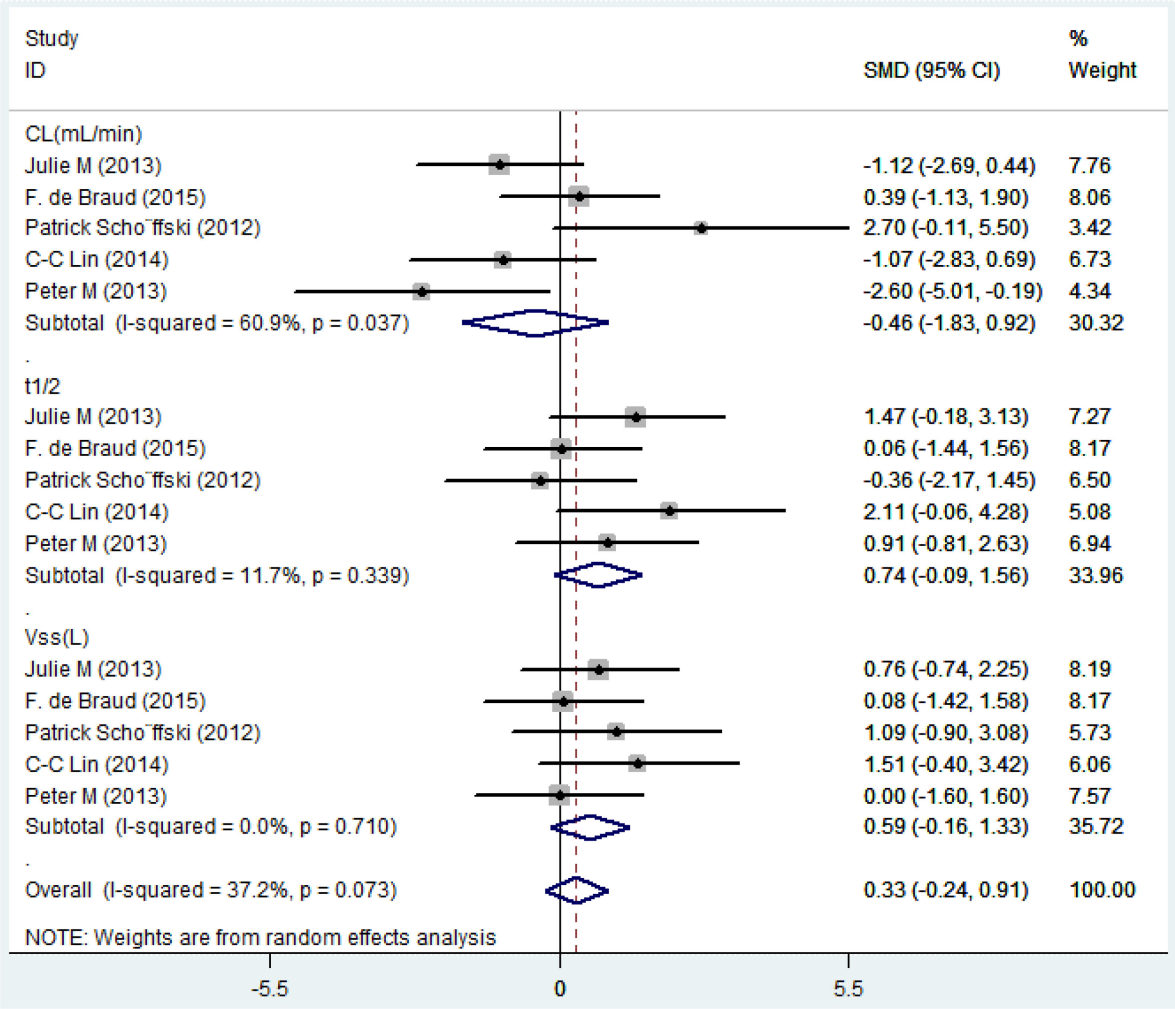
Forest plots of the pooled SMD for pharmacokinetic parameter by overall population.

Five eligible studies reported the pharmacokinetic parameters following one cycle of treatment. Among them, relevant studies that reported only mean value without standard deviation value were excluded from the pooled analysis. The overall estimate of SMD of pharmacokinetic parameters and 95% CIs from the individual studies was shown in **Figure 4** and **Table 7**. Due to the degree of heterogeneity (*I^2^* = 37.2%) remaining low and acceptable, the meta-analysis with the random-effects model was used to assess the outcome, and a random-effects model could be selected to perform heterogeneity tests for pooled results. As a result, the standard mean difference (SMD, 0.33; 95% CIs, -0.24-0.91) was presented through the meta-analysis, and assume that the test confirms that the SMD difference was not statistically significant (*Z* =1.13, *P* = 0.257). After using different doses of Plk1 inhibitors (the 100 mg low dosage cohort and the 200 mg high dosage cohort), there was no statistical difference in the total plasma clearance, terminal half-life and apparent volume of distribution at steady state.

**Table 7:** Meta-analysis of pharmacokinetic parameter by heterogeneity test.

Pharmacokinetic Parameter	*I^2^*	*P_I_*	*Z*	*P*
CL (mL/min)	60.90%	0.037	0.65	0.514
t_1/2_(h)	11.70%	0.339	1.75	0.081
Vss (L)	0.00%	0.710	1.55	0.121
Overall	37.20%	0.073	1.13	0.257

CL, total body clearance rate; t_1/2_, terminal half-life of the analyte in plasma.

Vss, apparent volume of distribution at steady state after an I.V. administration.

P, P-value for the variation in SMD attributable to significance test.

### Analysis of publication bias

**Figure 5:**
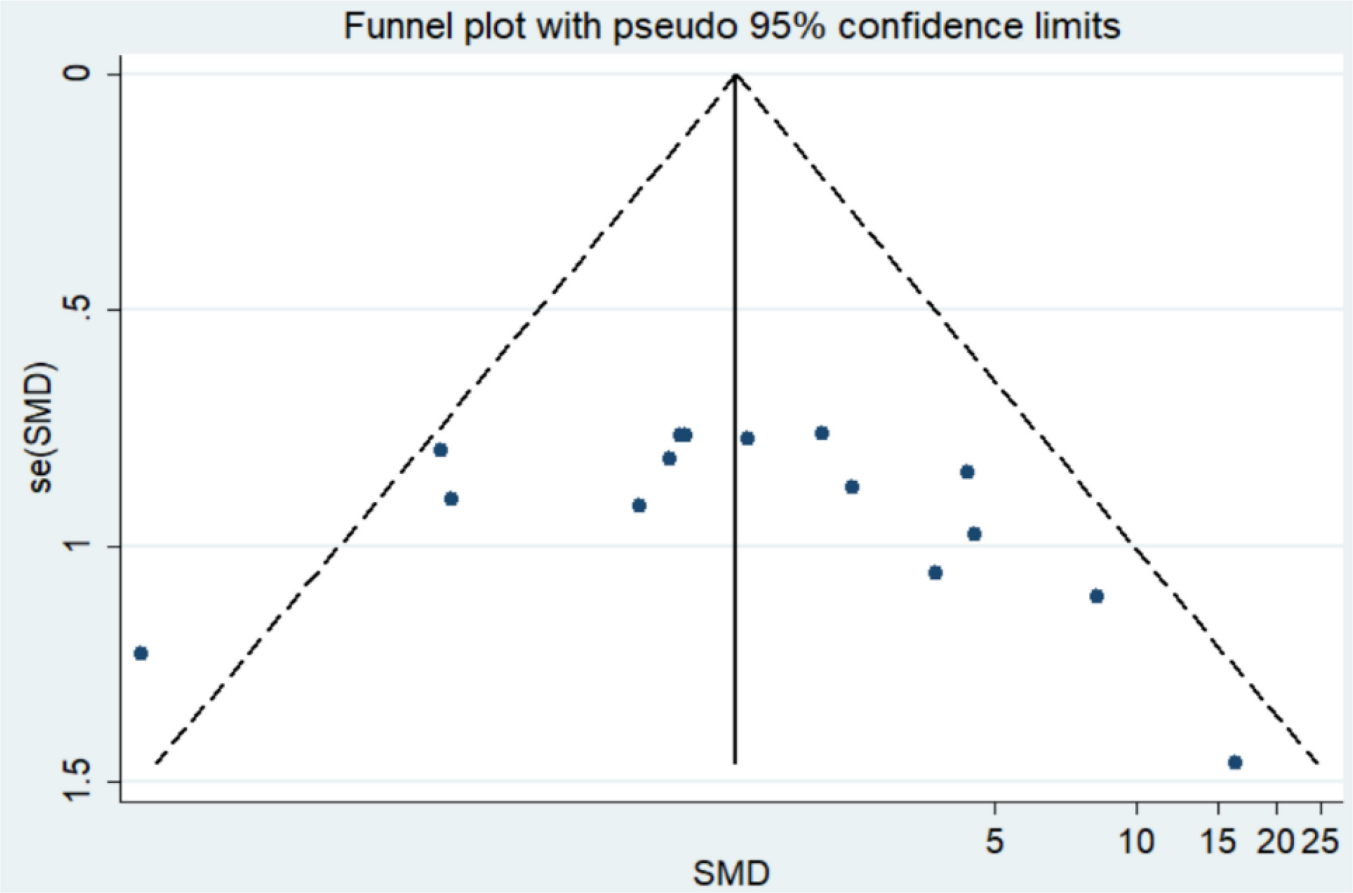
Funnel plot of asymmetry test of the publication bias of literatures.

We used Funnel plot and Egger’s regression asymmetry test to access the publication bias of literatures. Arrangement of data points did not reveal any evidence of obvious asymmetry. It was further confirmed by Egger’s linear regression asymmetry test for each outcome, and the results still did not show any evidence of publication bias (*t* = 0.85, *P* = 0.413) (**Figure 5** and **Table S2**).

## Discussion

Nowadays, many Plk1-related anti-cancer drugs are able to effectively kill tumor cells, but their unwanted toxicity to normal cells still restricts their clinical application (39, 40). To our best knowledge, this study is the first pharmacodynamic meta-analysis to evaluate the effectiveness and safety of the novel antitumor Plk1 inhibitors. In our study, all clinical trials included were published from 2008 to 2022, which reflected the popularity of Plk1 inhibitors in the past few years. Of all the 1256 patients, several types of cancers were mainly reported, such as metastatic pancreatic cancer, advanced solid tumors, acute myeloid leukemia, non-Hodgkin lymphoma, and pancreatic cancer.

Besides these, many trials in different kinds of cancers that are not eligible for inclusion are still under way. Although the results have not come out, it is possible that Plk1 inhibitors may work in patients against some certain types of tumors (41). Soon after their development, Plk1 inhibitors were used in hundreds of clinical trials that covered many different types of tumors (42). Since Plk1 inhibitors have been emerged as promising antitumor drugs, more and more efforts have been devoted to developing the relevant compounds and others as antineoplastic agents. Various inhibitors suppressing Plk1 kinases were involved in this study, mainly including BI 2536, Volasertib (BI 6727), Rigosertib (ON 01910.Na) and GSK461364A. Of note, Volasertib recently received clinical approval for human testing by the Food and Drug Administration (FDA) in 2020. (https://oncoheroes.com/press-releases-content/2020/10/14/volasertib-a-potential-new-treatment-for-rhabdomyosarcoma-receives-orphan-drug-designation-from-the-us-fda).

Will Plk1 inhibitors be a powerful and safe strategy for personalized cancer treatment in the future? According to our study, although there are certain adverse events in nervous system, digestive system and blood system, Plk1 inhibitors still worked well in safety and prolonged the OS of cancer patients. However, they failed to improve the PFS of cancer patients in this analysis. Apart from the overall level, we also analyzed the incidence of AEs based on tumor type and Plk1 inhibitor category. From these assessments, we summarized the conclusions of included overall level, and performed meta-analyses of subgroups for nervous system, blood system and digestive system. In terms of safety evaluation, it was found that whatever in the treatment of solid tumor or non-solid tumor, the incidence of AEs with Plk1 inhibitors was the most prominent in the hematological system. Among them, the incidence of AEs with BI 2536 and Volasertib (BI 6727) in the hematological system was relatively prominent, and the incidence of AEs of Rigosertib (ON 01910.Na) in the nervous system was also relatively obvious. These outcomes told us that in the selection process of Plk1 inhibitors for treating tumors, they might not be very suitable for hematological malignant tumors, such as neutropenia, acute myeloid leukemia and non-Hodgkin lymphoma. Especially for the most relevant adverse effect of Plk1 inhibitors, the neutropenia can be attributed to the transient inhibition of bone marrow precursor cell proliferation (43). Moreover, data from the present study have suggested that Plk1 inhibitors was associated with relevant neurotoxicity, which was possibly attributed to interactions with the tubulin cytoskeleton in nondividing differentiated cells. Of note, novel approaches to targeting key regulatory proteins for mitotic inhibition have the potential to overcome limitations of traditional antimitotic agents. It has been reported that, Rigosertib treatment of malignant tumor cells can cause severe mitotic spindle abnormalities and abnormal centrosome localization, G2-M cell cycle phase arrest and mitotic catastrophe, which eventually leads to apoptosis. In terms of mechanism of action, Rigosertib can interfere with the phosphoinositide 3-kinase (PI3K)/Akt, reactive oxygen species and Ras/Raf/Plk signaling pathways (17). Furthermore, based on the very good compliance observed, the administration of Plk1 inhibitors might also be very feasible in patients with non-specific tumors, respiratory system tumors, musculoskeletal system tumors, and urinary system tumors.

Pharmacokinetic evaluation showed that, when tumor patients were separately treated with low-dose 100 mg of Plk1 inhibitors and high-dose 200 mg of Plk1 inhibitors, there were no significant difference in total plasma clearance, terminal half-life, and apparent distribution volume of inhibitors in homeostasis. What surprised us even more was that even if the dose of the Plk1 inhibitors was doubled, it still could not significantly prolong the terminal half-life of the Plk1 inhibitor in plasma. In addition, it also could not markedly hinder the ability of liver and kidney organs to clear Plk1 inhibitors in plasma.

### Relation to prior work

Compared with the previous traditional meta-analysis, the use of indirect binary comparisons within this meta-analysis added additional information to the evidence on the efficacy, safety and pharmacokinetic efficacy of Plk1 inhibitors in the treatment of tumors, especially in determining the impact of Plk1 inhibitors on the treatment of tumors related to the nervous system, digestive system and blood circulation system. Our review included more trials and substantially more patients. Most importantly, for the first time, we refined the incidence of AEs to the respiratory system, urinary system, skin system, nervous system, facial features, digestive system (liver function, stomach, nausea or vomiting, appetite, abdomen), blood circulation system (anemia, neutropenia, leukopenia, thrombocytopenia), musculoskeletal system and nonspecific tissues and organs. Accordingly, it can be said that it is a relatively comprehensive systematic review and meta-analysis so far. In this review, we not only provide strategies for the selection of Plk1 inhibitors during clinical cancer treatment, but also make reasonable recommendations for the selection of dosages for their pharmacokinetic efficacy.

### Strengths of the study

The strength of our study is that we simultaneously used a meta-analysis to compare the outcomes of four different types of Plk1 inhibitors in solid versus non-solid tumor patients. Namely, the efficacy of Plk1 inhibitors was evaluated by PFS and OS. The safety of Plk1 inhibitors was assessed by the incidence of AEs in different systems, tissues or organs throughout the body. Subgroup analysis was conducted to evaluate not only the incidence of AEs in the nervous system, blood circulation system and digestive system during the treatment of solid tumors and non-solid tumors with Plk1 inhibitors, but also the incidence of AEs in the treatment of tumors with three Plk1 inhibitors (BI 2536, Volasertib (BI 6727) and Rigosertib (ON 01910.Na). Additionally, we compared the SMD of pharmacokinetic parameters (total plasma clearance, terminal half-life and apparent volume of distribution at steady state) to assess the pharmacokinetic effects of Plk1 inhibitors. More importantly, we included RCTs without language restrictions to avoid bias.

## Conclusions

This systematic review provides important information for weighing the potential benefits and harms of Plk1 inhibitors in the treatment of tumors. Key messages include: Plk1 inhibitors work better in improving the OS, and they are well tolerated, effective and safe in reducing the severity of illness while improving the quality of life, especially in patients with non-specific tumors, respiratory system tumors, musculoskeletal system tumors, and urinary system tumors. However, they fail to prolong the PFS. From the vertical whole level analysis, compared to other systems in the body, Plk1 inhibitors should be avoided as far as possible for the treatment of tumors related to the blood circulatory system, digestive system and nervous system, which were attributed to the intervention of Plk1 inhibitors associated with an increased risk of AEs in these systems. The toxicity caused by immunotherapy should be carefully considered. Conversely, a horizontal comparison of three different types of Plk1 inhibitors suggested that Rigosertib (ON 01910.Na) might be relatively suitable for the treatment of tumors associated with the digestive system, while Volasertib (BI 6727) might be even less suitable for the treatment of tumors associated with the blood circulation system. Additionally, in the dose selection of Plk1 inhibitors, the low dose of 100 mg should be preferred, and meanwhile, it can also ensure the pharmacokinetic efficacy that is indistinguishable from the high dose of 200 mg. Due to the limited quality and quantity of the included studies, more high-quality studies are needed to validate the above conclusions. This review provided key evidence for the relevant frontiers in oncology rapid recommendations, which used additional context and methodology to generate recommendations for clinical practice.

## Data availability statement

The original contributions presented in the study are included in the article/**Supplementary Material**. Further inquiries can be directed to the corresponding authors.

## Author contributions

Conception and design: XW, MS and CH. Development of methodology: XW, MS and CH. Acquisition of data: XW, MS, GRJ, QY, GHJ. Analysis and interpretation of data: XW, MS and CH. Writing, review, and/or revision of the manuscript: XW, MS, CH, XJ and ZS. Administrative, technical, or material support: XW, MS and ZS. Study supervision: MS, XJ and ZS. All authors contributed to the article and approved the submitted version.
