# Supplementary material for: Effectiveness, safety and pharmacokinetics of Polo-like kinase 1 inhibitors in tumor therapy: A systematic review and meta-analysis
Source: Front Oncol. 2023 Feb 9;13:1062885. doi: 10.3389/fonc.2023.1062885 (PMC9947705; doi:10.3389/fonc.2023.1062885)
Supplement: Supplementary file 1 [file DataSheet_1.docx]

**Supporting Information:**

**Effectiveness, Safety and Pharmacokinetics of** **Polo-like kinase 1 (Plk1) Inhibitors in Tumor Therapy: A Systematic Review and Meta-analysis**

*Xiao Wei ^1,a^, Mingzhu Song ^1,a,^*^#^ *, Chan Huang ^1,a^, Qiao Yu ^a^,Guirong Jiang ^a^, Guanghao Jin ^a^, Xibiao Jia ^c,#^ and Zheng Shi ^b,#^*

*^a.^ School of Preclinical Medicine,* *Chengdu University, Chengdu, 610106, P. R. China*

*^b.^ Clinical Genetics Laboratory，Clinical Medical college & Affiliated hospital of Chengdu University, Chengdu University, Chengdu,* *610106, P. R. China*

*^c.^ Key Laboratory of Ministry of Education of birth defects and related Maternal and*

*Child Diseases, West China second Hospital, Sichuan University, 610066, P. R. China*

1. Xiao Wei, Mingzhu Song and Chan Huang contributed equally to this work, should be regarded as co-author of the first authors.

# Corresponding authors at: School of Preclinical Medicine, Chengdu University, Chengdu, 610106, P. R. China (M.Z. Song); Key Laboratory of Ministry of Education of birth defects and related Maternal and Child Diseases, West China second Hospital, Sichuan University, 610066, P. R. China (X.B. Jia); Clinical Genetics Laboratory, Clinical Medical college & Affiliated hospital of Chengdu University, Chengdu University, Chengdu, 610106, P. R. China (Z. Shi)

E-mail: [songmingzhu@cdu.edu.cn](mailto:songmingzhu@cdu.edu.cn) (M.Z. Song), [381918820@qq.com](mailto:381918820@qq.com) (X.B. Jia), [drshiz1002@hotmail.com](mailto:drshiz1002@hotmail.com) (Z. Shi)


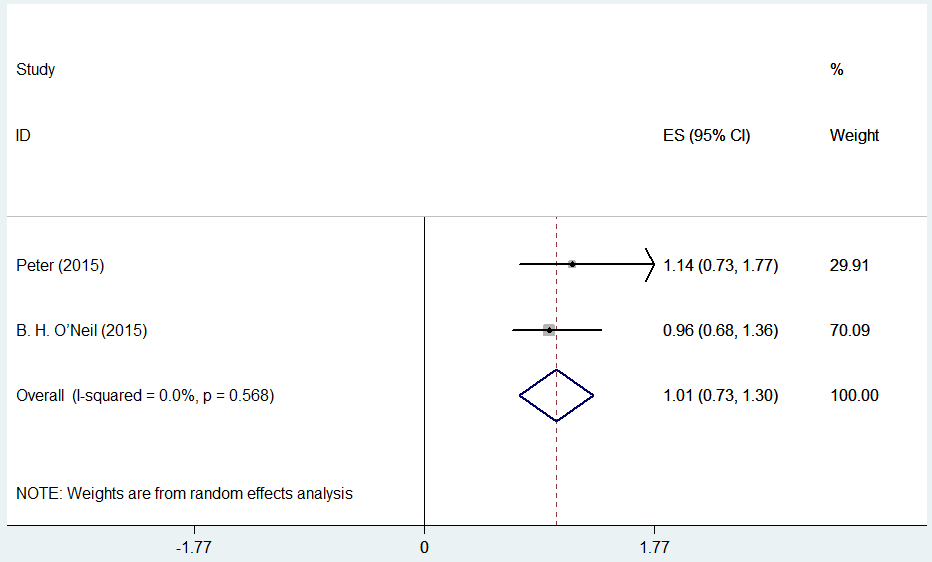


**Figure S1.** Forest plots of the pooled ES for PFS by overall population.


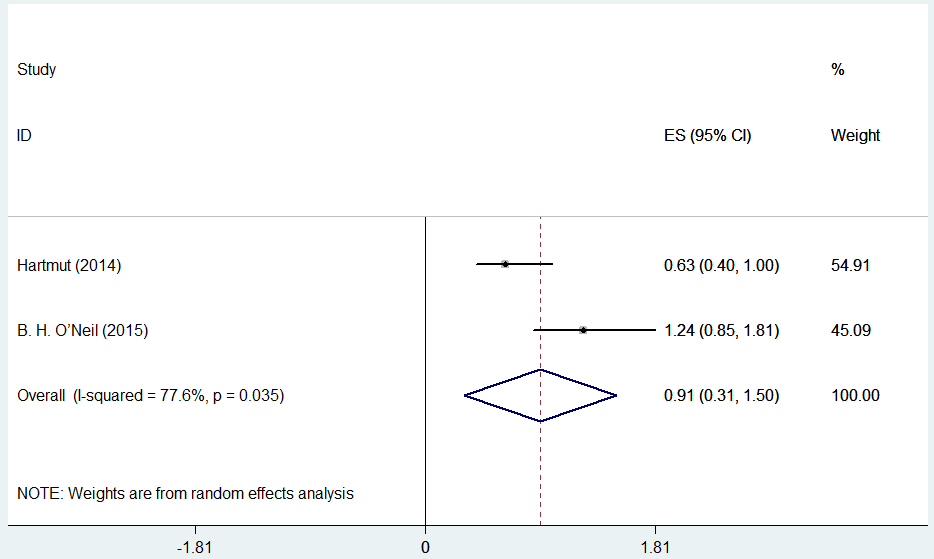


**Figure S2.** Forest plots of the pooled ES for OS by overall population.


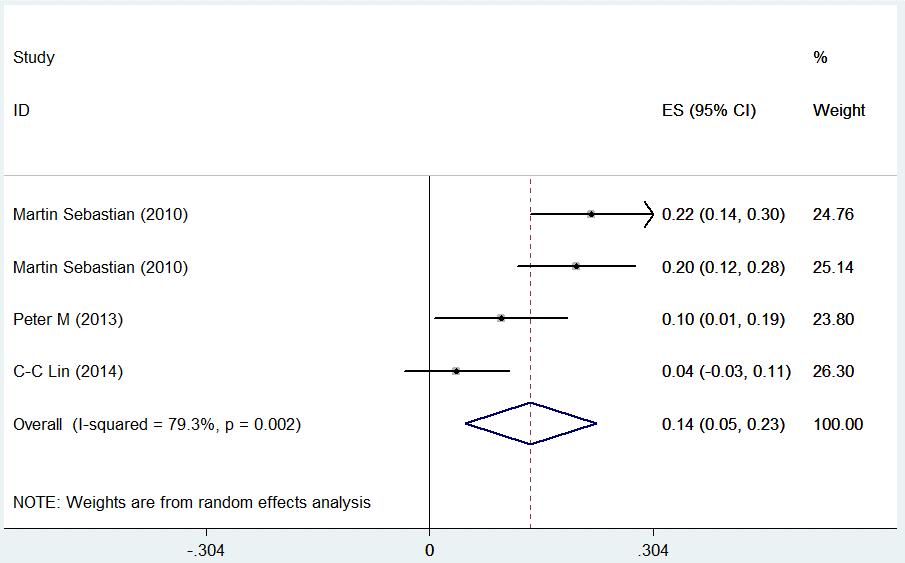


**Figure S3.** The incidence of adverse events in respiratory system.


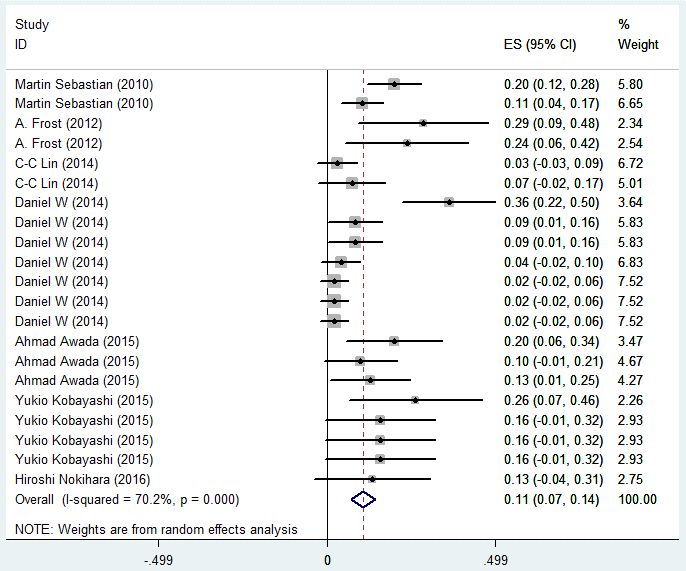


**Figure S4.** The incidence of adverse events in urinary system.


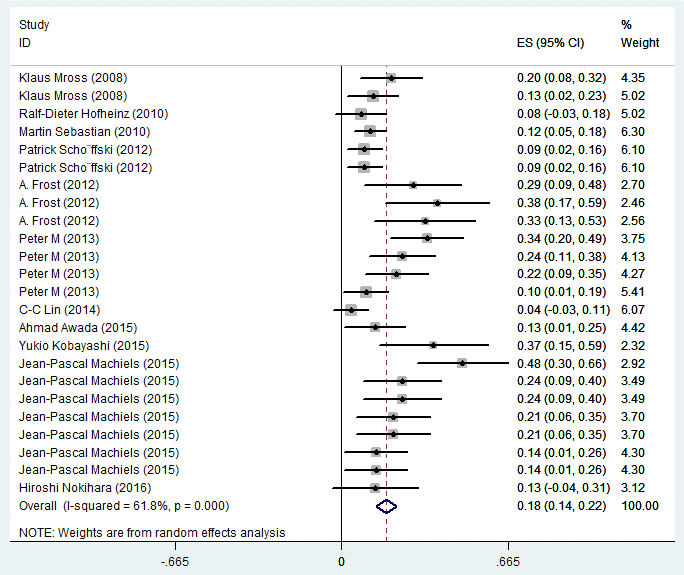


**Figure S5.** The incidence of adverse events in skin system.


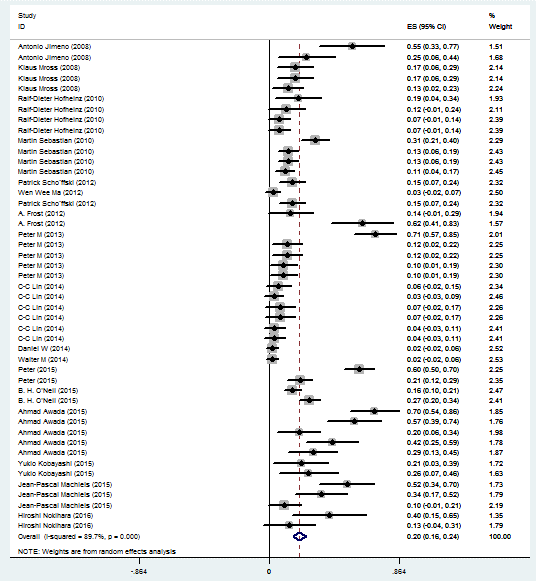


**Figure S6.** The incidence of adverse events in nervous system.


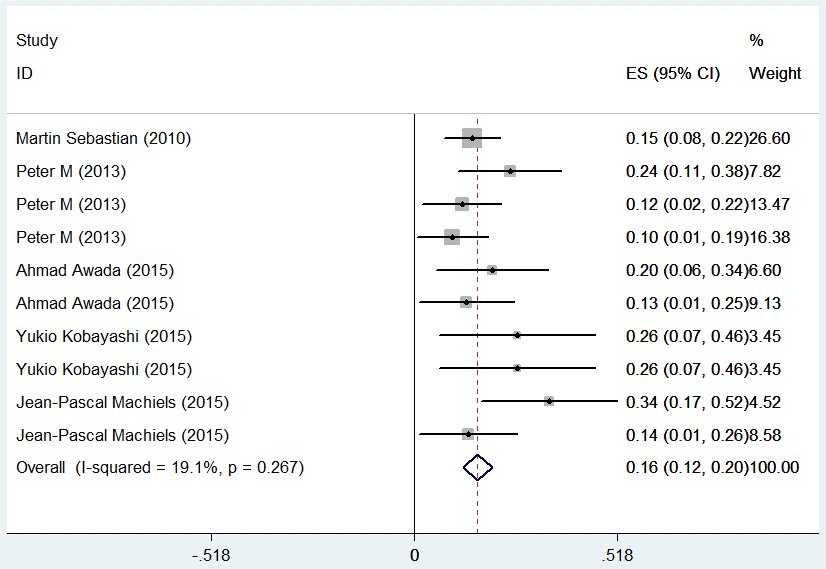


**Figure S7.** The incidence of adverse events in facial features.


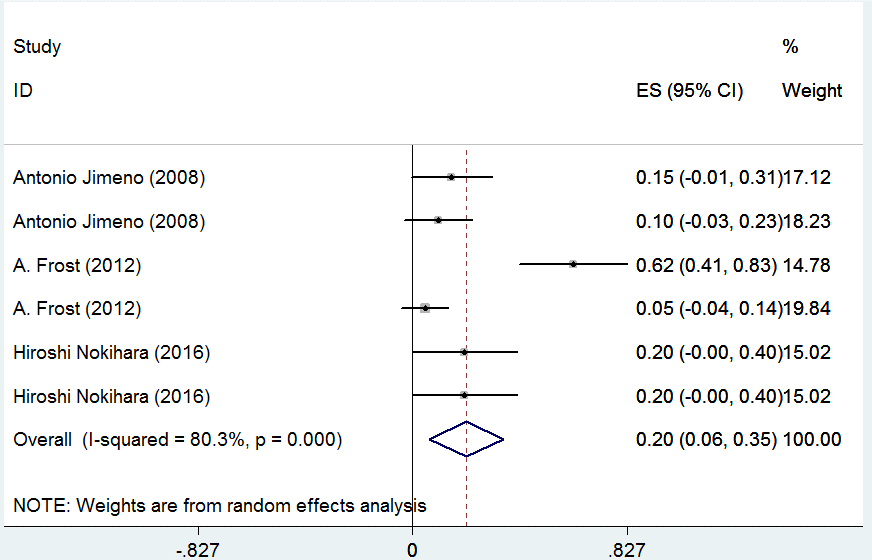


**Figure S8.** The incidence of adverse events in liver function.


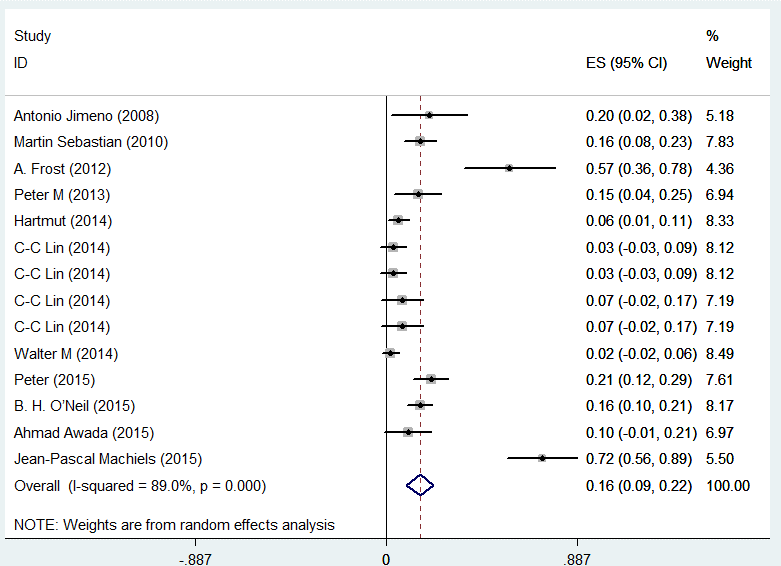


**Figure S9.** The incidence of adverse events in stomach function.


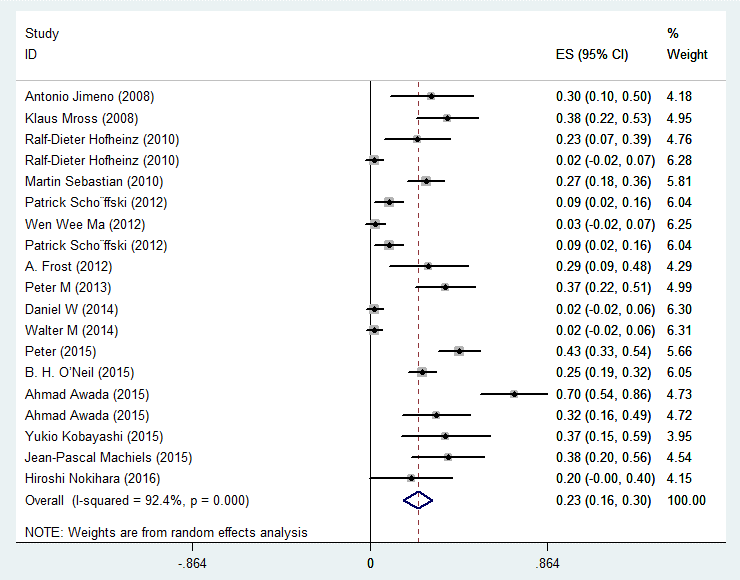


**Figure S10.** The incidence of adverse events in nausea or vomiting.


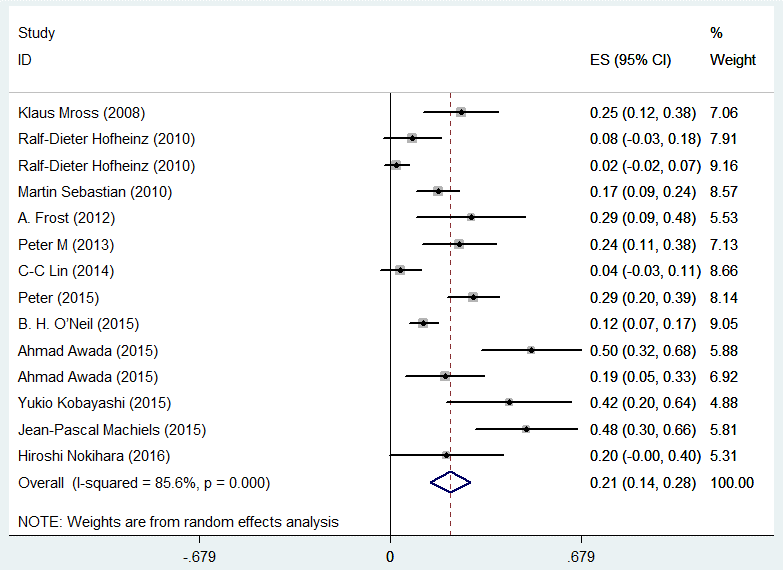


**Figure S11.** The incidence of adverse events in appetite.


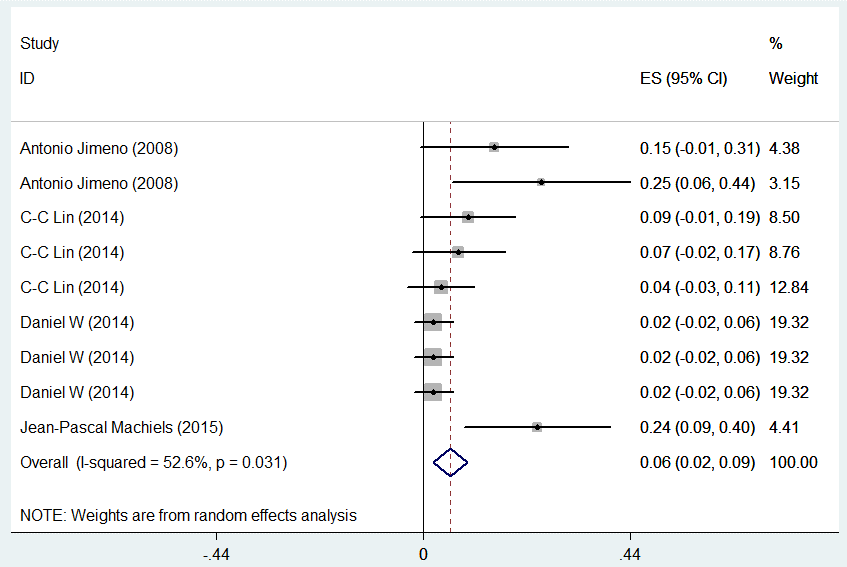


**Figure S12.** The incidence of adverse events in abdomen.


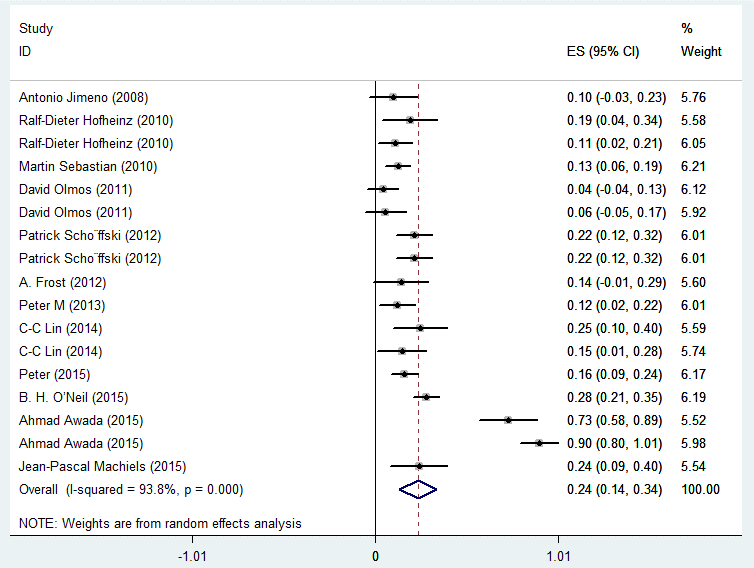


**Figure S13.** The incidence of adverse events in anemia.


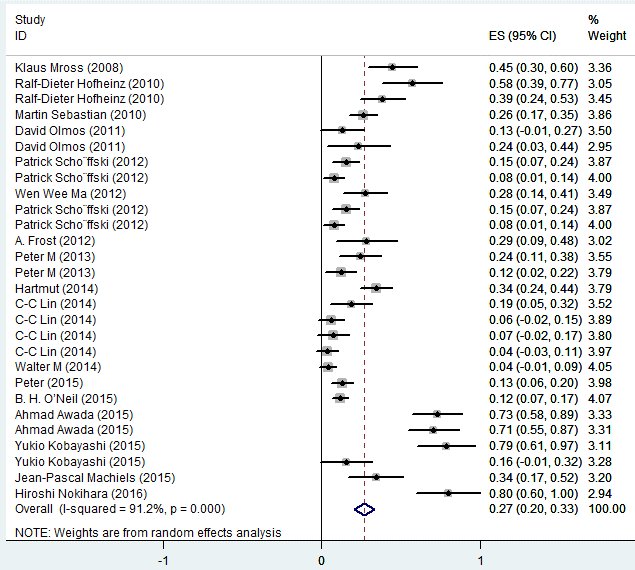


**Figure S14.** The incidence of adverse events in neutropenia.


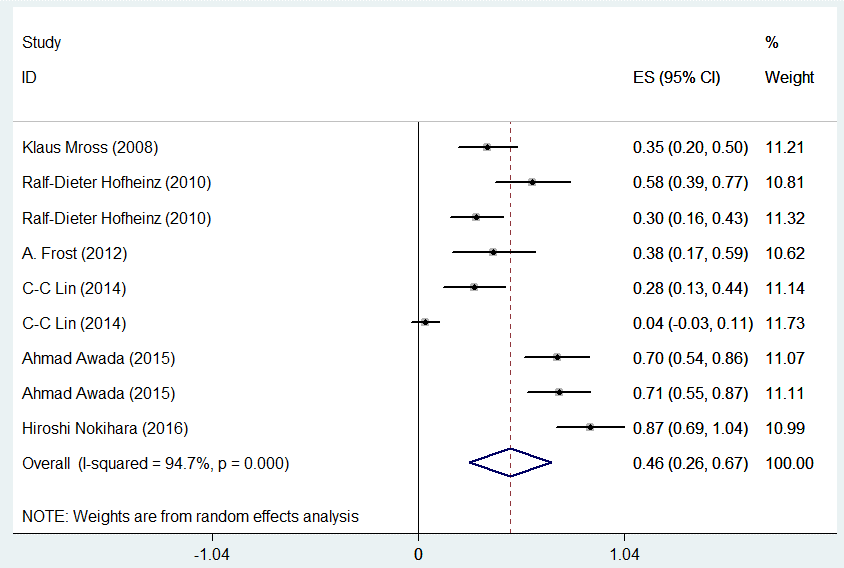


**Figure S15.** The incidence of adverse events in leukopenia.


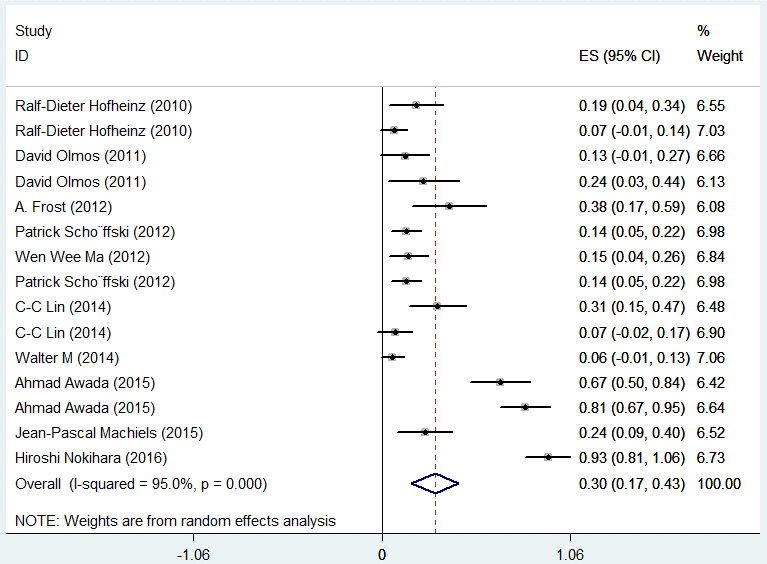


**Figure S16.** The incidence of adverse events in thrombocytopenia.


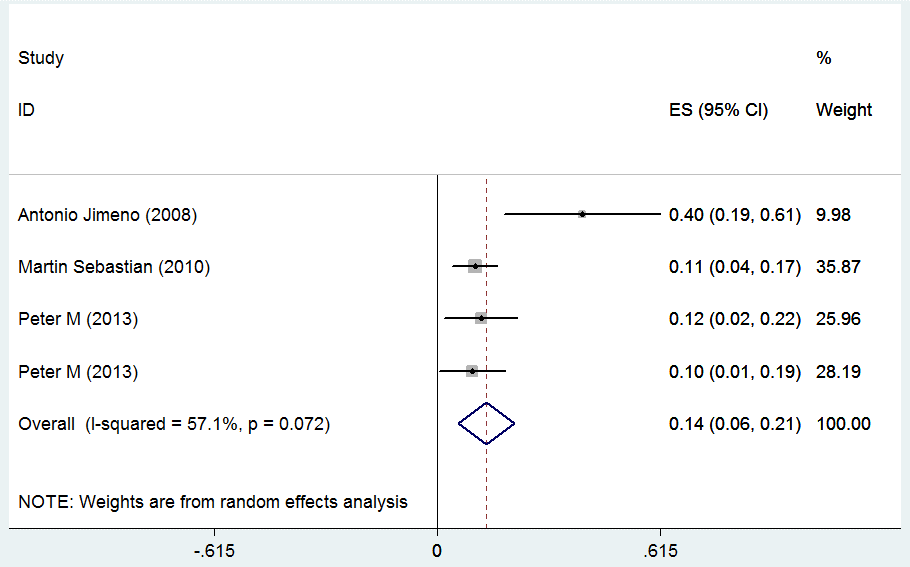


**Figure S17.** The incidence of adverse events in musculoskeletal function.


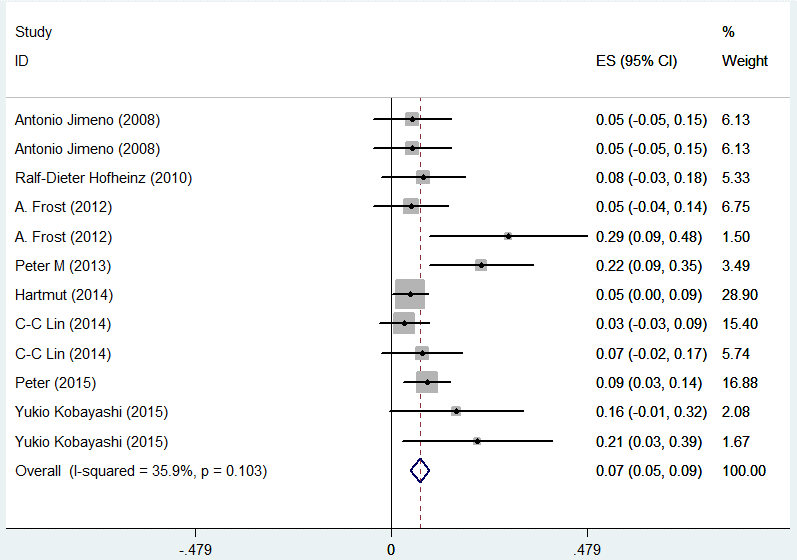


**Figure S18.** The incidence of adverse events in non-specific tissues and organs.

**Table S1.** Egger’s regression asymmetry test to access the publication bias of literatures.

| Std_Eff | Coef. | Std. Err. | t | P>\|t\| | [95% Conf. Interval] |
| --- | --- | --- | --- | --- | --- |
| slope | -1.105 | 0.503 | -2.20 | 0.043 | [-2.172 -.0387] |
| bias | 2.674 | 0.989 | 2.70 | 0.016 | [0.577 4.770] |

**Table S2.** Egger’s regression asymmetry test to access the publication bias of literatures.

| Std_Eff | Coef. | Std. Err. | t | P>\|t\| | [95% Conf. Interval] |
| --- | --- | --- | --- | --- | --- |
| slope | -1.122 | 1.741 | -0.64 | 0.531 | [-4.882 2.639] |
| bias | 1.654 | 1.954 | 0.85 | 0.413 | [-2.567 5.875] |
