# Supplementary figures and images for: Effectiveness, safety and pharmacokinetics of Polo-like kinase 1 inhibitors in tumor therapy: A systematic review and meta-analysis
Source: Front Oncol. 2023 Feb 9;13:1062885. doi: 10.3389/fonc.2023.1062885 (PMC9947705; doi:10.3389/fonc.2023.1062885)

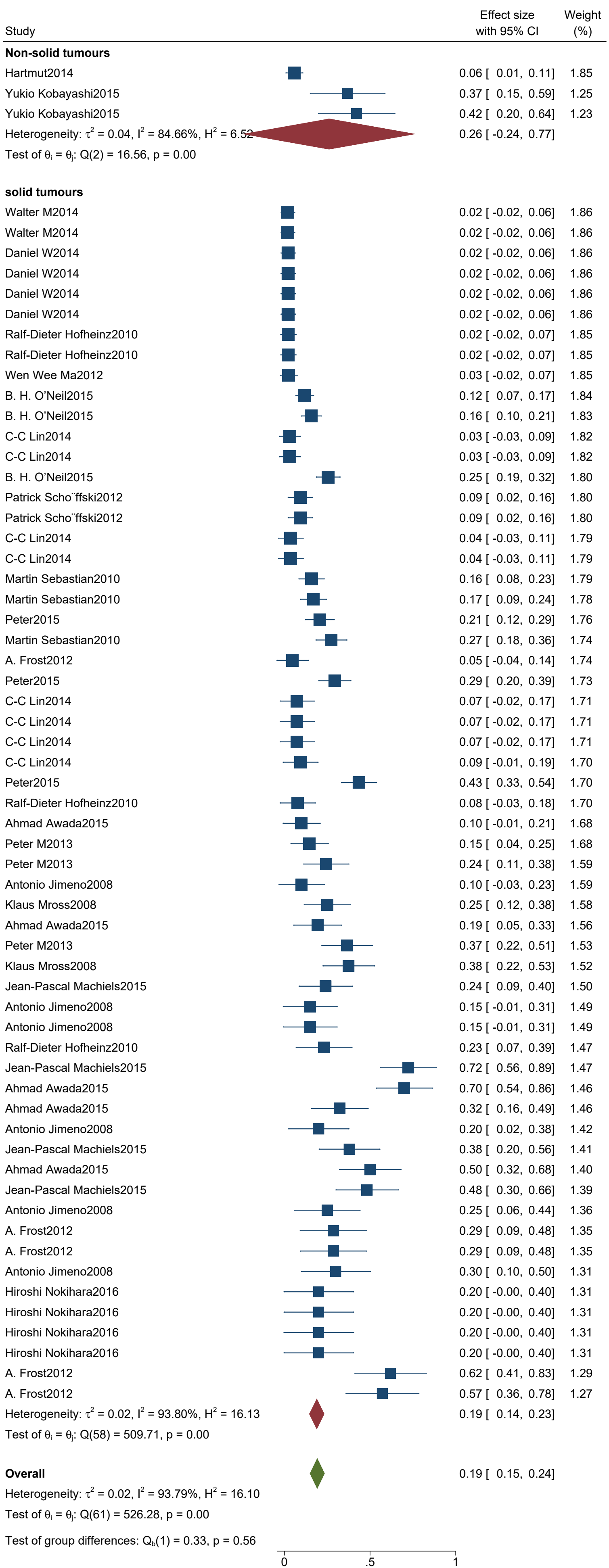

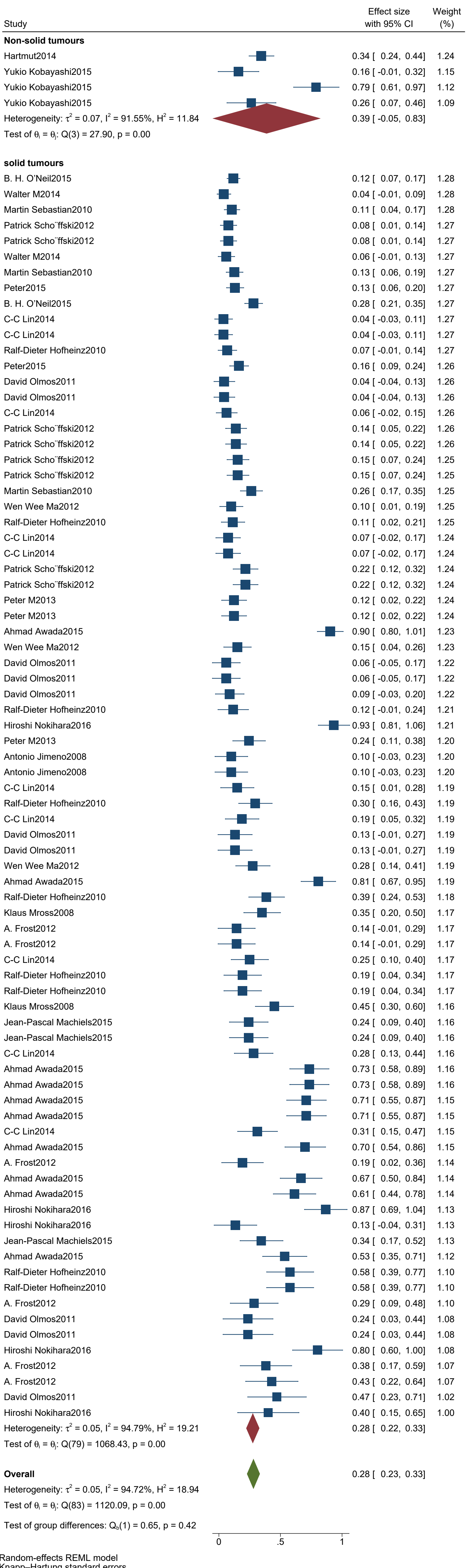

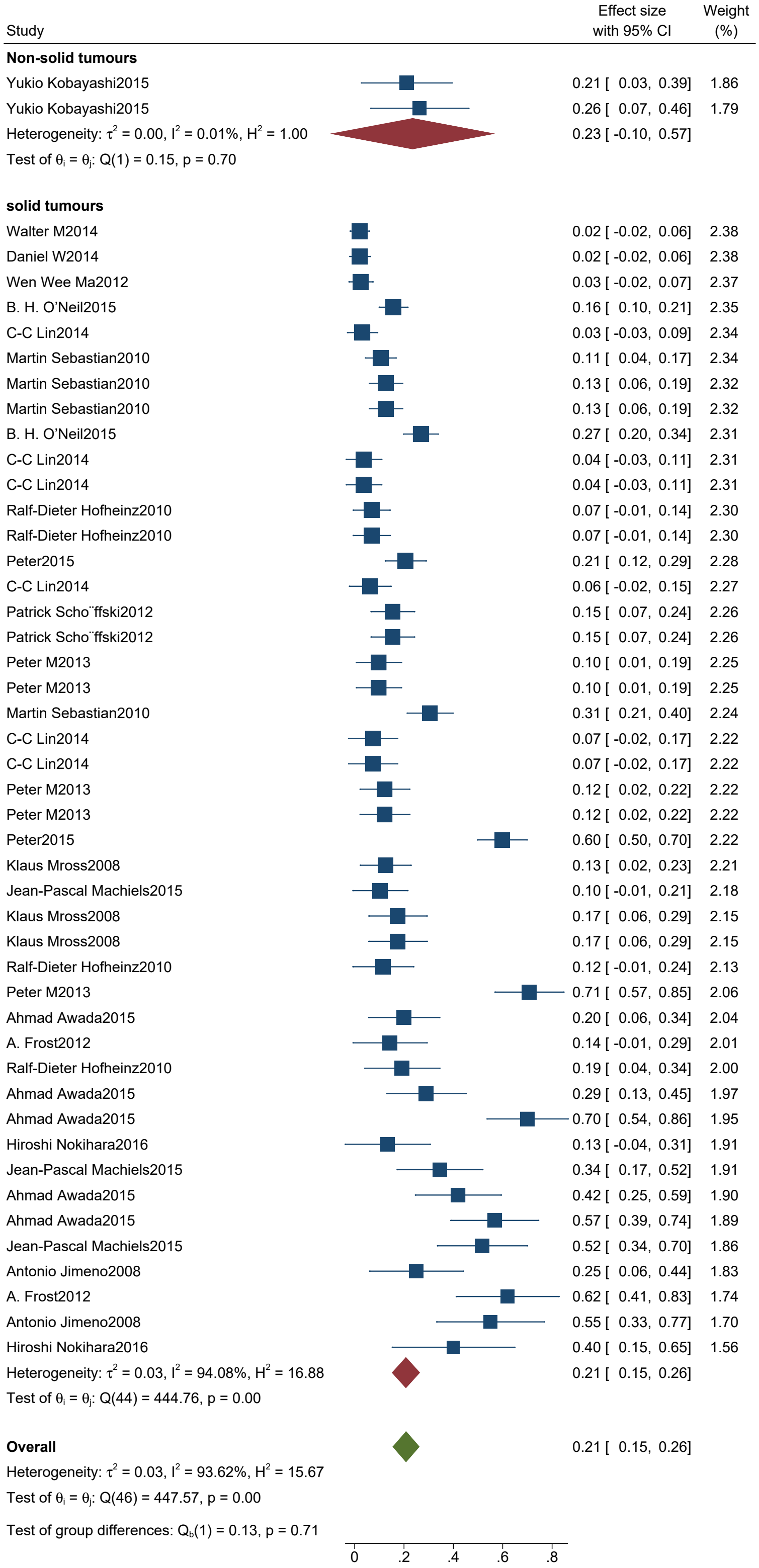

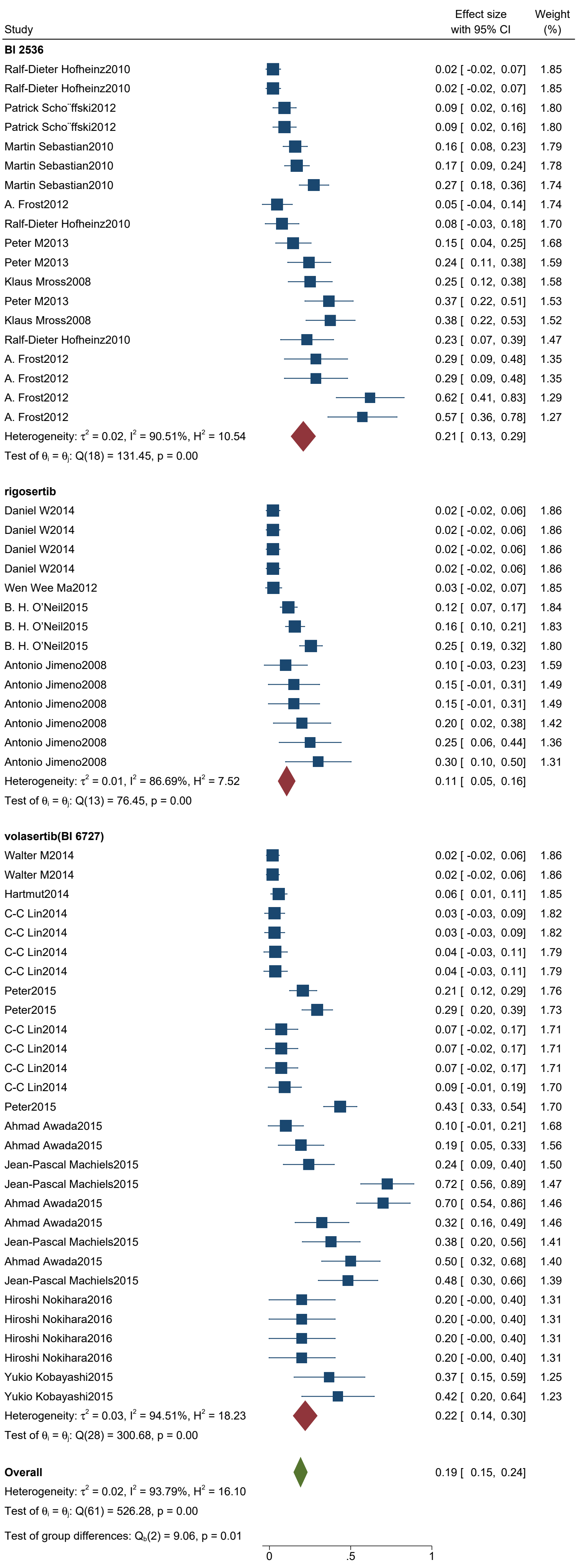

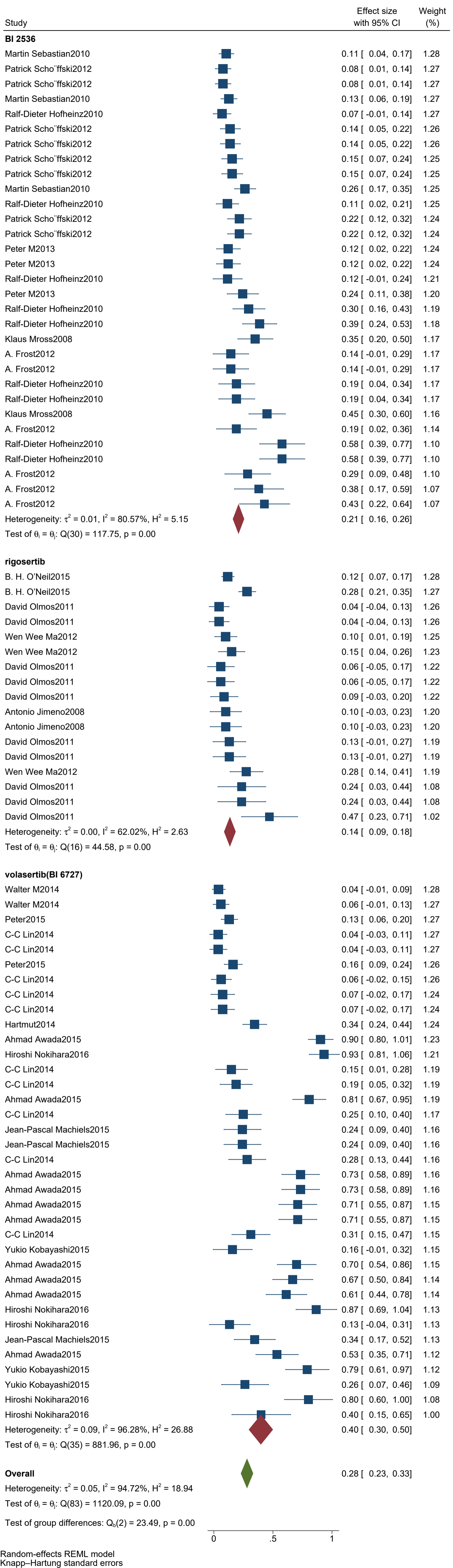

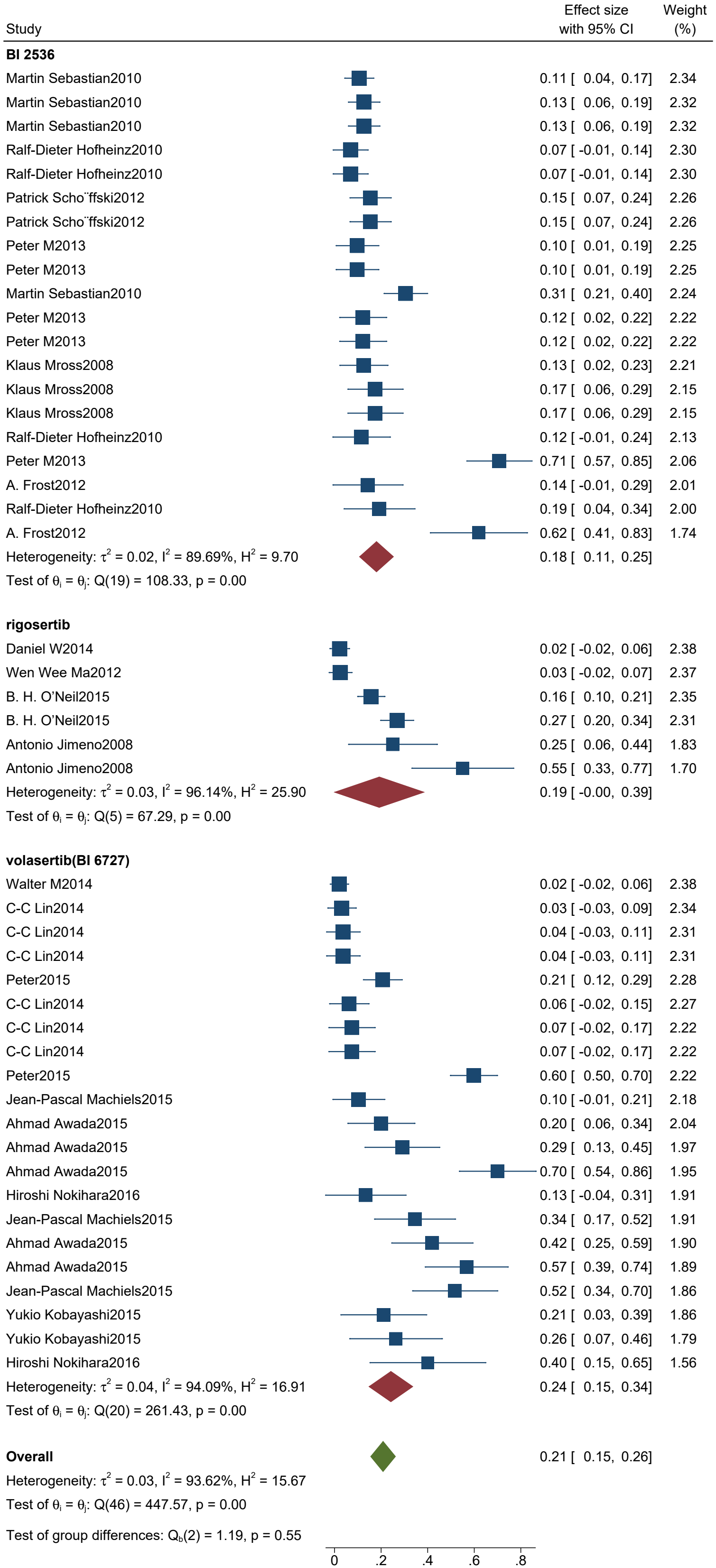

Supplement: Supplementary file 2 [file DataSheet_2.pdf]
